# Rare Variant Association Testing for Next-Generation Sequencing Data via Hierarchical Clustering

**DOI:** 10.1159/000346022

**Published:** 2013-04-11

**Authors:** Ioanna Tachmazidou, Andrew Morris, Eleftheria Zeggini

**Affiliations:** ^a^Wellcome Trust Sanger Institute, Hinxton, University of Oxford, Oxford, UK; ^b^Wellcome Trust Centre for Human Genetics, University of Oxford, Oxford, UK

**Keywords:** Allele match kernel, Genetic similarity, Next-generation sequencing, Single nucleotide polymorphism

## Abstract

**Objectives:**

It is thought that a proportion of the genetic susceptibility to complex diseases is due to low-frequency and rare variants. Next-generation sequencing in large populations facilitates the detection of rare variant associations to disease risk. In order to achieve adequate power to detect association at low-frequency and rare variants, locus-specific statistical methods are being developed that combine information across variants within a functional unit and test for association with this enriched signal through so-called burden tests.

**Methods:**

We propose a hierarchical clustering approach and a similarity kernel-based association test for continuous phenotypes. This method clusters individuals into groups, within which samples are assumed to be genetically similar, and subsequently tests the group effects among the different clusters.

**Results:**

The power of this approach is comparable to that of collapsing methods when causal variants have the same direction of effect, but its power is significantly higher compared to burden tests when both protective and risk variants are present in the region of interest. Overall, we observe that the Sequence Kernel Association Test (SKAT) is the most powerful approach under the allelic architectures considered.

**Conclusions:**

In our overall comparison, we find the analytical framework within which SKAT operates to yield higher power and to control type I error appropriately.

## Introduction

Large-scale genome-wide association studies have been successful in identifying common variants influencing complex traits. Although these findings have improved our understanding of the genetic basis of many complex traits, for most of the traits they explain only a fraction of heritability. This observation supports the long established idea that low-frequency and rare variants play an important role in common diseases. Thanks to recent advances in sequencing and genotyping technology and to large collaborative efforts that study human sequence variation and the allelic architecture of disease, such as the 1000 Genomes Project (www.1000genomes.org) [1] and the UK10K project (www.uk10k.org), we are now able to study the contribution of low-frequency and rare variants in complex traits. The different strategies in place today to search for low-frequency and rare variants affecting complex traits largely fall under 3 categories: genome-wide association studies using dense genotyping platforms (for example, Illumina 2.5M and the exome chip), imputation, and next-generation sequencing. Here we focus on the analysis of data generated by next-generation sequencing, although the methods presented here are applicable to data from the other categories. We also discuss ways of extending the methods to allow for genotype uncertainty, which can arise from imputation or low-coverage next-generation sequencing.

Single-point analysis of low-frequency (typically defined as those with minor allele frequency (MAF) between 1 and 5%) or rare variants (those with MAF <1%) is under-powered, as not enough copies of the minor allele are observed. An alternative approach is to use methods that combine information across multiple low-frequency or rare variant sites within a region, which can be a gene or any other functional genomic region. Currently, there are more than thirty methods developed for rare variant burden analysis, which use different approaches to combine information across multiple variants within the region of interest. One group of tests consists of collapsing methods based on summary statistics (the Cohort Allelic Sums Test [2], Combined Multivariate and Collapsing method [3], Weighted Sum Test [4], Variable-Threshold approach [5]), another group of tests are based on similarities among individual sequences (the Kernel-Based Association Test [6], Sequence Kernel Association Test [7] – SKAT), while other methods are based on regression models that use collapsed sets of variants and other factors as predictors (collapsing using proportion of rare variants [8], adaptive sums [9], LASSO or ridge regression-based approaches [10]). Collapsing tests aggregate information across multiple variants into a single quantity, which is then used to test for association of disease with an accumulation of rare minor alleles, whereas methods based on similarities among individual sequences are multi-marker tests that combine single-variant test statistics. Methods that use the regression model have the advantage that they can easily adjust for covariates and can easily handle both a continuous and case-control outcome.

Collapsing methods vary in the way they collapse low-frequency/rare variants and in their chosen test statistic. A popular burden test was proposed by Morris and Zeggini [8], which models a continuous or binary phenotype as a function of the proportion of low-frequency/rare variants at which an individual carries a minor allele within a regression framework. This model implicitly assumes that all collapsed variants are associated with disease, and that they have the same direction of effect, which can be either deleterious or protective. Moreover, it is not robust to linkage disequilibrium (LD), as correlation between the collapsed variants can lead to inﬂation of the test statistic. However, we do not expect this to be a worry for the analysis of rare variants, as we expect there to be relatively limited LD between them, because they are likely to have occurred relatively recently in the ancestry of the population. A different kind of approach is SKAT [7], which is based on a weighted linear kernel function that measures the genetic similarity among the study samples. A continuous or binary phenotype is also modelled within a regression framework, but as variants are not collapsed together, it allows each variant to have its own direction and magnitude of effect or even no effect.

Another approach that uses similarities among individual sequences is the Kernel-Based Association Test (KBAT) proposed by Mukhopadhyay et al. [6]. This method focuses on a case-control phenotype and measures the genetic similarity for each pair within cases and controls at each low-frequency/rare variant by counting the common alleles between the genotypes of two individuals. This scoring scheme is based on the Allele Match (AM) kernel, and the similarity scores between individuals are modelled via a one-way ANOVA model that tests whether the group effects are the same for cases and controls. KBAT is a nonparametric test that makes no assumptions about the direction of individual SNP effects and is robust to LD. Asimit et al. [11] extended KBAT to account for genotype uncertainty of sequence-derived data in a software called AMELIA (Allele Matching Empirical Locus-specific Integrated Association test), but the method is only applicable for a case/control outcome. Incorporating sequence-derived variant calling and genotype probability uncertainty into the analysis of sequence-based datasets is important, because in sequencing datasets there is uncertainty surrounding variant calling (which is more pronounced in sequencing projects at low depths), while genotype misclassification is more of a concern compared to datasets arising from genotyping platforms. These types of risks can lead to loss of power for subsequent association studies, which is illustrated in the results of Asimit et al. [11], according to which AMELIA is consistently more powerful than its unweighted equivalent KBAT.

Here we focus on a continuous phenotype and we explore a hierarchical clustering and kernel-based association test. Specifically, we suggest using the AM kernel to cluster individuals into disjoint clusters and test if the means of the phenotypes within each cluster are the same via ANOVA. We use a simple hierarchical clustering algorithm, where our distance matrix is one minus a standardized AM similarity score matrix. To investigate the performance of the proposed method and its power and type I error, we performed an extensive simulation study with varying sample sizes, different magnitudes of genetic signal and different allelic architectures and compared it to two of the most popularly used rare variant analysis methods. We find that its power is comparable to that of the collapsing test based on the proportion of low-frequency/rare variants when causal variants have the same direction of effect, but its power is almost double than that of the burden test when both protective and risk variants exist. SKAT is, however, the clear winner in both allelic architecture scenarios.

## Methods

### Collapsing Methods

Morris and Zeggini [8] introduced a collapsing method based on the proportion of low-frequency/rare variants at which an individual carries a minor allele. If *n*_*i*_ is the number of low-frequency/rare variants present in the *i*-th individual with a quantitative phenotype *y*_*i*_, then for the analysis of a quantitative trait this collapsing test is implemented as

(1) $$y_{i}=\beta +\lambda \frac{r_{i}}{n_{i}}+\beta x_{i}+\varepsilon_{i},$$

where *λ* is the increase in the phenotype value for an individual carrying minor alleles at *r*_*i*_ low-frequency/rare loci compared to an individual carrying none, ***β*** denote regression coefficients for a vector of covariates ***x***_*i*_, and *ε*_*i*_ ∼ *N*(0,*σ*_*E*_). Association of an accumulation of low-frequency/rare variants with the phenotype can be tested by examining the null hypothesis *H*_0_: *λ* = 0 by using a likelihood ratio test. This method has been implemented in the GRANVIL software (http://www.well.ox.ac.uk/GRANVIL).

### Sequence Kernel Association Test

SKAT [7] is implemented within a multiple regression framework, where phenotype *y*_*i*_ of sample *i* is modelled as

(2) $$y_{i}=\alpha_{0}+\alpha x_{i}+\beta g_{i}+\varepsilon_{i},$$

where ***β*** is a vector of regression coefficients for genotypes ***g***_*i*_ = (*g*_*i*__1_, …, *g*_*ip*_) that individual *i* carries at *p* variants within the region, and *g*_*ij*_ = 0, 1, or 2 is the number of copies of the minor allele. Moreover, ***α*** are regression coefficients for a vector of covariates ***x***_*i*_, and *ε*_*i*_ ∼ *N*(0,*σ*_*E*_).

SKAT assumes that each *β*_*j*_ follows an arbitrary distribution centered at zero with variance *ω*_*j*_*τ,* where *τ* is a variance component and *ω*_*j*_ is a prespecified weight for variant *j*. To test for association of the variants with the phenotype, SKAT tests *H*_0_: *τ* = 0 by employing a variance component score statistic given by

(3) $$Q={\left(y-\overset{‸}{\mu }\right)}^{\prime }K\left(y-\overset{‸}{\mu }\right),$$

where $\overset{‸}{\mu }$ is the predicted mean of *y* under *H*_0_. For a study of *n* individuals, *K* is an *n* × *n* matrix whose (*i, i*′) element is given by the weighted kernel function

(2) $$K\left(G_{i},G_{i^{\prime }}\right)=\sum_{j=1}^{p}\omega_{j}G_{ij}G_{i^{\prime }j},$$

where *G* is an *n* × *p* matrix with (*i*, *j*) element being the genotype of variant *j* of individual *i*.

SKAT puts more weight on rare variants and non-zero weight to variants with MAF 1-5%. This is achieved by assuming that $\sqrt{\left(\omega_{j}\right)}$ has magnitude given by the Beta density function Beta(MAF_*j*_; *α*_1_, *α*_2_) evaluated at the MAF of variant *j* and with parameters *α*_1_ = 1 and *α*_2_ = 25. Other choices for weights are also discussed in Wu et al. [7].

Under *H*_0_, *Q* follows a mixture of χ^2^ distributions (please refer to Wu et al. [7] for an explanation of how this is approximated). SKAT can be applied to all variants across the region of interest, but we have limited it to low-frequency or rare variants to make results comparable to the rest of the methods we tested.

### A Hierarchical Clustering and Kernel-Based Association Test

We propose a simple hierarchical clustering approach, where the individuals in the study are allocated to distinct clusters according to their genotype similarity across the region of interest. The similarity measure we employ is the AM kernel score suggested by Mukhopadhyay et al. [6]. The AM kernel is the number of alleles shared between two individuals at a single locus. It is ﬂexible, as it makes no assumptions on the risk allele of each variant.

We then use the ANOVA model to test if the means of the phenotypes within each cluster differ significantly. Specifically, for any pair (*i*, *j*) of individuals *i* ≠ *j* in the study with sample size *n* and for each low-frequency/rare variant *k* in the region of interest spanning *p* low-frequency/rare variants, the AM kernel assigns a similarity score

(4) $$h^{k}\left(g_{\left(i\right)}^{k},g_{\left(j\right)}^{k}\right)\{\begin{array}{ll} 4, & if\;g_{\left(i\right)}^{k}=g_{\left(j\right)}^{k}\\ 2, & if\;g_{\left(i\right)}^{k}=1,g_{\left(j\right)}^{k}\in \left\{0,2\right\}\;\text{or}\;g_{\left(j\right)}^{k}=1,g_{\left(i\right)}^{k}\in \left\{0,2\right\},\\ 0, & \text{otherwise} \end{array}$$

where $g_{\left(i\right)}^{k}$ and $g_{\left(j\right)}^{k}$ are the number of minor allele individuals *i* and *j* carry at low-frequency/rare variant *k*. For any pair (*i*, *j*) of individuals *i* ≠ *j*, the AM score across the region of interest is simply the sum of the single-SNV AM scores, i.e.

$$h\left(g_{\left(i\right)},g_{j}\right)=\sum_{k=1}^{p}h^{k}\left(g_{\left(i\right)}^{k},g_{\left(j\right)}^{k}\right).$$

We perform a hierarchical cluster analysis using the AM score to group individuals according to their genotype similarity, or rather according to their genotype dissimilarity, which is represented by the *n* × *nD* matrix with (*i*, *j*) element being *d*_(__*ij*__)_ = 1 − *h*(*g*_(__*i*__)_, *g*_(__*j*__)_)/4*p*. Hierarchical clustering works by initially assigning each individual to their own cluster and then the algorithm proceeds iteratively, at each stage joining the two most similar clusters, continuing until there is just a single cluster. At each stage, distances between clusters are recomputed according to the particular clustering method being used. We use Ward's hierarchical clustering, which minimizes the information loss (defined by Ward in terms of an error sum-of-squares criterion) associated with each grouping. Hierarchical cluster analysis can be readily implemented by using the standard statistics package R, and returns a dendogram, i.e. a tree whose branches represent the different individuals, so that genetically ‘close’ individuals appear in the same or neighbouring branches. A caveat when using hierarchical clustering is that in order to allocate samples to groups, the user needs to ‘cut’ the tree at a prespecified height, or in other words, to prespecify the number of clusters.

If the number of clusters is specified to *L*, the study samples are allocated to these *L* distinct clusters according to their position in the dendogram. We then model the phenotype *y*_*li*_ of individual *i* = 1, …, *n* belonging to cluster *l* = 1, …, *L* using a one-way ANOVA model as

(5) $$y_{li}=\mu +\alpha_{1}+\varepsilon_{i},$$

where *µ* is the general effect of individuals, *α*_*l*_ is the cluster-specific treatment effect, and *ε*_*i*_ is the error component. If individuals' phenotypes between clusters are similar, then *α*_1_ = ··· = *α*_*L*_, and therefore *H*_0_ for testing disease association is *α*_1_ = ··· = *α*_*L*_ = 0.

The within-group sum of squares is

$$SSW=\sum_{l=1}^{L}\sum_{i=1}^{nj}{\left(y_{li}-{\bar{U}}_{l}\right)}^{2},\;\text{where}\;\;{\bar{U}}_{l}=\sum_{i=1}^{nj}y_{li}/n_{l}$$

and *n*_*l*_ denotes the number of individuals in group *l*. The between-group sum of squares is

$$SSB=\sum_{l=1}^{L}n_{l}{\left({\bar{U}}_{l}-\bar{U}\right)}^{2},\;\text{where}\;\;\bar{U}=\left({\bar{U}}_{1}+\cdots +{\bar{U}}_{L}\right)/n.$$

To test association with disease, the ANOVA statistic is given by

(6) $$F=\frac{SSB/\left(L-1\right)}{SSW/\left(n-L\right)},$$

which follows an *F*-distribution with (*L* − 1) and (*n* − *L*) degrees of freedom.

We vary the number of clusters from 1 to 20, and we repeat the ANOVA modelling step, which is very fast, and report the maximum test statistic. We find that using an upper threshold of 20 for the possible number of clusters is sufficient to capture the genetic diversity of the study samples, by examining the average number of clusters that correspond to the maximum test statistic in our simulation study (e.g. for a simulation scenario with 3,000 individuals, the mean and median value was 8.7 and 6, respectively, across 1,000 replications). To assign significance to the association test, we perform 10,000 permutations of the phenotype to individuals and we repeat the ANOVA step with the number of clusters ranging from 1 to 20, without the need to repeat the hierarchical clustering. The p value of significance of the association test is the proportion of permutations that resulted in a maximum test statistic bigger than or equal to the original maximum test statistic.

It is possible to down-weight variants that are not thought to be disease-associated and up-weight variants that are believed to be disease-associated, so that the contribution of the latter to the AM score across the region of interest is higher. We have therefore implemented a version of the model that incorporates weights. In this instance, the AM score across the region of interest is given by

$$h^{\prime }\left(g_{\left(i\right)},g_{\left(j\right)}\right)=\sum_{k=1}^{p}\omega_{k}h^{k}\left(g_{\left(i\right)}^{k},g_{\left(j\right)}^{k}\right),$$

where *ω*_*k*_ is the weight of variant *k*, and the dissimilarity matrix *D*′ has (*i*, *j*) element equal to

$${d^{\prime }}_{\left(ij\right)}=1-h^{\prime }\left(g_{\left(i\right)},g_{\left(j\right)}\right)/\sum_{k=1}^{p}4\omega_{k}.$$

The un-weighted version of the proposed approach can be viewed as the weighted version with weights *ω*_*k*_ = 1 for all *k* = 1, …, *p*. Different weighing schemes are possible, and in our simulation study, we used the one proposed by Wu et al. [7], where $\sqrt{\left(\omega_{k}\right)}$ has a magnitude given by the Beta density function Beta(MAF_*k*_; 1, 25) evaluated at the MAF of variant *k* (please see above), so that more weight is assigned to rarer variants.

The method is implemented in an R- and C-based software called KATE (Kernel Association Test Extended). KATE is freely available at http://www.sanger.ac.uk/resources/software/kate/.

### Simulation Study

We conducted an extensive simulation study to examine the type I error and power of the proposed method, and to compare its performance to other popular rare variant methods under different allelic architectures. Haplotype data were simulated under the null model of no genetic association for 2*N* individuals by using the HAPGEN2 software [12], so that the allele frequency spectrum and LD structure is the same with the 1000 Genomes haplotype data (Phase I interim June 2011 release in NCBI build 37 coordinates, 89 GBR samples). We limited the simulated region to a representative gene on chromosome 21 (*PFKL* with coordinates 45,719,934 and 45,747,259 base pairs) with 143 polymorphic SNPs (39 SNPs have MAF <5%, and 17 SNPs have MAF <1%).

Haplotypes were randomly paired to form *N* individuals, where *N* = 1,000, 2,000, 3,000. Causal variants were randomly selected such that their individual MAF did not exceed 0.02 and their total MAF did not exceed 0.05, as implemented in Asimit et al. [11]. A continuous phenotype *y*_*i*_ was assigned to each individual *i* according to a linear model

(7) $$y_{i}=\beta_{\text{l}}g_{i\text{l}}^{c}+\cdot \cdot +\beta_{s}g_{is}^{c}+\varepsilon_{i},$$

where *s* is the number of causal variants, $g_{i1}^{c},\ldots ,g_{is}^{c}$ are the genotypes of the causal variants for individual *i*, and *ε*_*i*_ follows a standard normal distribution. Each causal variant *k* = 1, …, *s* had an effect *β*_*k*_ on the phenotype. To examine the type I error, all regression coefficients *β*_*k*_ were set to 0, which translated to the null disease model of no association. We simulated scenarios where all causal variants were deleterious, and other scenarios where half of the causal variants were deleterious and the rest were protective. For the deleterious variants, *β*_*k*_ was set to 1.2, 2 and 3, whereas for the protective variants, *β*_*k*_ = −1.2, −2, −3. We also considered a scenario where the effect size was not fixed, but it varied with the MAF of the causal variant. Following Wu et al. [7], we set the magnitude of each *β*_*k*_ as |*β*_*k*_| = 0.4|log_10_MAF_*k*_| to allow rarer causal variants to have larger effects. As the individual MAF cannot exceed 0.02, the smallest individual effect is |*β*_*k*_| = 0.68. Table 1 summarizes the different parameter settings used for the effect sizes.

Using this procedure we generated 1,000 replicates under each simulation scenario. We subsequently used the simulated data with KATE, GRANVIL and SKAT. For each simulation scenario where the data have been simulated with a genetic signal (regression coefficients *β*_*k*_ > 0), the power of each test is calculated as the proportion of replicates for which the test yielded a p value of less than a 5% significance threshold. For data simulated under the null disease model of no association, we calculate the false-positive rate of each test as the proportion of replicates for which the test yielded a p value of less than 5%.

## Results

Table 2 reports the type I error rate together with the corresponding 95% confidence intervals for KATE, GRANVIL and SKAT. We observe that all methods control the type I error rate well at 5%, with perhaps the exception of KATE in the scenario where weights are assumed to have a Beta distribution, where the type I error rate is closer to 6%. The 95% confidence interval for a 5% error rate is (3.6, 6.4)%, so KATE and GRANVIL are not always consistent with a 5% error rate.

Tables 3 and 4 show the empirical power of the three methods for the different sample sizes and under different magnitudes of effect sizes when causal variants are assumed to be only risk-increasing and when causal variants are also allowed to be protective, respectively. Overall, we observe that SKAT is the most powerful approach under any allelic architecture considered. When causal variants have the same direction of effect, GRANVIL tends to perform better than KATE by a factor of approximately 16% for weaker effects, and by 1-8% for stronger effects. However, the power of GRANVIL sharply decreases when the direction of effect differs among the causal variants, while KATE's performance is not affected. In this scenario, KATE's power is between 32 and 50% higher than that of GRANVIL, depending on the magnitude of the association signal. Moreover, the use of weights for KATE increases its power by 6-8%. As expected, power increases with increasing sample size and magnitude of effect for all methods. The increase in power with sample size is more pronounced for KATE.

These results are based on a single gene region (gene *PFKL* in chromosome 21). This gene is representative of chromosome 21 in terms of size and number of variants with MAF <5 or <1%, and we therefore believe that our conclusions are robust to the choice of gene. However, all three methods can be implemented genome-wide, which would give a more complete picture of their relative power.

## Discussion

Powerful allele-matching approaches for the analysis of rare variants have been proposed [6,13], but their relative power for quantitative traits remains unclear. We have presented KATE as a method for low-frequency/rare variants association analysis that exploits the AM kernel to model a continuous trait. This method does not make assumptions about the directionality of effect and is valid when the variants in the region of interest are in LD. KATE can be implemented using both common and low-frequency/rare variants, which is also true for SKAT, but we used only the low-frequency/rare variants to make results comparable to the results from collapsing methods. Using an extensive simulation study, we find that KATE has similar power to GRANVIL when all causal variants are designed to have the same direction of effect. In the presence of both deleterious and protective variants, the power of GRANVIL sharply decreases, whereas KATE's performance is not affected.

In terms of computational speed, both GRANVIL and SKAT are very fast, as they do not require permutation testing. In contrast, KATE relies on permutation to assign significance. KATE is reasonably fast for a small number of samples (*N* = 1,000), but becomes computational intensive as the number of samples increases (*N* = 3,000). We used 10,000 permutations to assign significance in the simulation study presented here, although an adaptive number of permutations is also possible and could reduce KATE's running time. In this adaptive design, the default number of permutations can be 1,000 and increased to 10,000 only if the p value is less than a pre-specified threshold, as implemented by Asimit et al. [11].

It has been shown that the use of weights can increase the power of rare variant methods. We illustrate this by implementing the weights used in Wu et al. [7], which up-weight rare variants. However, any type of weighting scheme can be easily implemented with KATE, such as weights derived from functional annotation tools. Moreover, it is straightforward to adjust the model for genotype uncertainty for imputed variants by incorporating genotype probabilities in the AM scoring system. Variant quality scores available for sequence-derived datasets can also easily be adjusted for in KATE in the same way as illustrated in AMELIA [11].

It is straightforward to extend KATE to include covariates in order to increase the precision of comparisons between groups by accounting for variation on important prognostic variables, and to adjust comparisons between groups for imbalances in important prognostic variables between these groups. This can be achieved by replacing ANOVA with ANCOVA (analysis of covariance), which is a technique that combines analysis of variance and regression analysis. In this situation, the permutation procedure needs to be adapted, so that at each permutation and for each pair of individuals we exchange not only their phenotypes but also their covariate values.

In our overall comparison, we find the analytical framework within which SKAT operates to yield higher power and to control type I error appropriately.

## Figures and Tables

**Table 1:** Effect sizes used in the simulation study for each causal variant *k*

Deleterious effects only	1.2	2	3	0.4|log_10_MAF_*k*_|
Deleterious and protective effects	–1.2, 1.2	–2, 2	–3, 3	−0.4|log_10_MAF_*k*_|, 0.4|log_10_MAF_*k*_|

In the presence of different directions of effect (second line of the table), the two effect sizes reported are evenly distributed among the causal variants.			

**Table 2:** Type I error rates and their respective confidence intervals (in percentage) for a threshold of 5% for the different methods under the various simulation scenarios considered

*N*	KATE		GRANVIL	SKAT
	weights = 1	weights ~ beta		
1,000	5.2 (3.8, 6.6)	5.9 (4.4, 7.4)	5.1 (3.7, 6.5)	4.5 (3.2, 5.8)
2,000	4.9 (3.6, 6.2)	5.9 (4.4, 7.4)	4.6 (3.3, 5.9)	4.9 (3.6, 6.2)
3,000	5.1 (3.7, 6.5)	5.7 (4.3, 7.1)	5.7 (4.3, 7.1)	4.9 (3.6, 6.2)

**Table 3:** Power comparisons for the different methods under the various effect and sample sizes considered when causal variants have the same direction of effect

	KATE		GRANVIL	SKAT
	weights = 1	weights ~ beta		
*N = 1,000*			
|beta|=1.2	0.671	0.713	0.794	1
|beta|=2.0	0.935	0.954	0.953	1
|beta|=3.0	0.969	0.974	0.984	1
|beta| ∝ MAF	0.320	0.395	0.558	0.971

*N = 2,000*			
|beta|=1.2	0.887	0.899	0.937	1
|beta|=2.0	0.986	0.985	0.981	1
|beta|=3.0	0.988	0.991	0.998	1
|beta| ∝ MAF	0.568	0.648	0.817	1

*N = 3,000*			
|beta|=1.2	0.958	0.965	0.975	1
|beta|=2.0	0.982	0.987	0.996	1
|beta|=3.0	0.996	0.998	0.999	1
|beta| ∝ MAF	0.759	0.799	0.919	1

**Table 4:** Power comparisons for the different methods under the various effect and sample sizes considered when there exist both protective and risk variants

	KATE		GRANVIL	SKAT
	weights = 1	weights ~ beta		
*N = 1,000*			
|beta|=1.2	0.625	0.665	0.220	0.999
|beta|=2.0	0.921	0.934	0.367	1
|beta|=3.0	0.969	0.973	0.480	1
|beta| ∝ MAF	0.331	0.393	0.147	0.970

*N = 2,000*			
|beta|=1.2	0.891	0.909	0.380	0.999
|beta|=2.0	0.982	0.983	0.505	1
|beta|=3.0	0.991	0.994	0.621	1
|beta| ∝ MAF	0.577	0.636	0.213	0.998

*N = 3,000*			
|beta|=1.2	0.952	0.960	0.426	1
|beta|=2.0	0.987	0.990	0.599	1
|beta|=3.0	0.988	0.991	0.672	1
|beta| ∝ MAF	0.769	0.816	0.292	1
